# The gut mycobiome and inter-kingdom microbial networks are linked to COPD severity in lung cancer patients

**DOI:** 10.1038/s41598-026-47296-x

**Published:** 2026-04-30

**Authors:** György Szklenarik, David Dora, Sára Szincsak, Clement Kojo Acquah, Arghajit Biswas, Mátyás Horváth, Gabriella Galffy, Zoltan Lohinai

**Affiliations:** 1 Translational Medicine Institute, Semmelweis University, Tűzoltó utca 37-47, Budapest, Budapest, 1094 Hungary; 2 Department of Anatomy, Histology and Embryology, Semmelweis University, Budapest, Hungary; 3 County Hospital of Torokbalint, Torokbalint, Hungary

**Keywords:** Gut mycobiome, COPD, Cross-kingdom networks, Fungal ecology, Gut-lung axis, Diseases, Microbiology

## Abstract

Chronic obstructive pulmonary disease (COPD) is increasingly recognized as a systemic disorder affecting host–microbiome interactions beyond the airways. Although bacterial alterations in COPD have been documented, the gut mycobiome and its ecological integration with bacterial communities remain unexplored. In this study, we profiled the gut mycobiome of 61 non-small-cell lung cancer (NSCLC) patients stratified by COPD severity using ITS2 sequencing and analyzed 47 overlapping patients with available metagenomic data to construct cross-kingdom bacterial–fungal networks. Alpha diversity, assessed by Shannon, Simpson, and Chao1 indices, did not differ significantly between patients with and without severe COPD. Partial least squares discriminant analysis (PLS-DA) revealed partial separation of the two groups, with COPD severity explaining 6% of overall compositional variance (R²=0.06, *p* = 0.058). COPD-severe patients exhibited a significantly reduced Ascomycota/Basidiomycota ratio (*p* = 0.039) and lower relative abundance of *Mucoromycota*. Analysis of compositions of microbiomes (ANCOM) identified *Myrothecium* and *Lasiodiplodia crassispora* enriched in severe COPD, while *Helotiales_unclassified* and *Phallus atrovolvatus* were more abundant in non-severe cases. Fungal co-occurrence networks demonstrated reduced connectivity and modularity in severe COPD compared with non-severe COPD. Cross-kingdom analyses integrating bacterial genera revealed strengthened *Candida*–*Enterococcus*/*Clostridium* hubs and weakened *Faecalibacterium*/*Roseburia*–yeast associations in severe disease. Keystone analysis showed increased centrality for *Candida*, *Aspergillus*, *Enterococcus*, and *Clostridium*, and decreased centrality for *Akkermansia* and *Roseburia*. A compositional balance classifier achieved high discriminatory power (AUC = 0.88) in distinguishing COPD-severe from non-severe patients. These findings indicate that COPD severity is not characterized by major diversity loss but by guild-specific compositional shifts and extensive network rewiring, favoring oxygen-tolerant, opportunistic taxa over short-chain fatty acid–associated commensals.

## Introduction

Chronic obstructive pulmonary disease (COPD) is one of the three leading causes of death, affecting around 10% of the population, with an expected increase by 23% from 2020 to 2050, including females by 47.1%^1,2^. COPD is characterized by exposure to inhaled particulate matter, including smoking or air pollutants, in combination with genetic, developmental, and social factors^3^. The identification of “treatable traits” is crucial through the identification of “phenotypes” and “endotypes”. Therefore, a better understanding of molecular mechanisms might reveal novel associations beyond our current understanding of gene-environment interactions^4,5^.

The gut-lung axis may help extend our current understanding of the dynamics of maintenance, repair, and cumulative tissue injury, as well as aging, in the pathology of COPD. The gastrointestinal and respiratory tracts share similar cellular components, but their distribution varies to support organ-specific functions. Mucosa-associated lymphoid tissue (MALT) comprises gut-associated lymphoid tissue (GALT) and inducible bronchus-associated lymphoid tissue (iBALT). The microbiome contributes to mucosal and systemic immune homeostasis through the mononuclear phagocyte system (MPS), which mediates host–microbe crosstalk^6^. A notable example of the mutual changes in the gut-lung axis microbiome is the increase in *Streptococcus* species in both organs among smokers^7^. *Lactobacillus* was shown to play an immunomodulatory role in the management of patients with chronic respiratory diseases by balancing lung immunity and promoting respiratory health through the bidirectional gut-lung axis^8^. Regarding metabolites, short-chain fatty acids (SCFAs) produced by anaerobic bacteria in the gut have a beneficial effect on the immune system’s response to infections, including COPD^9^. Other studies have raised the possibility of further microbiota dysbiosis, with alterations in the gut microbiota, specifically in the Fusobacteriaceae, *Prevotellaceae*, and *Bacteroidaceae* genera, and a reduced *Prevotella*/*Moraxella* ratio in the lungs^10^. Further research has shown that the abundance of *Rothia*, *Romboutsia*, *Intestinibacter*, *Escherichia*, *Lachnospira*ceae, *Aerococcus*, and *Fusobacterium* increased in COPD, but that *Bacteroides*, *Roseburia*, *Lachnospira*, *Ruminococcaceae*, and *Lachnoclostridium* decreased^11^.

Of the millions of species of fungi that exist, around 300 are present in the human body. The mycobiome, the fungal component of the community, comprises less than 1% of the human gut microbiota. According to the Human Microbiome Project, the fungal communities were characterized by a high prevalence of *Saccharomyces*, *Malassezia*, and *Candida*, in addition to *S. cerevisiae*, *M. restricta*, and *C. albicans*. The *Ascomycota* and *Basidiomycota* phyla are almost exclusively present in the human intestinal tract, whereas other phyla are mostly found in pathological conditions^12^. The significance of the *Ascomycota/Basidiomycota* (A/B) ratio and *Candida spp*. is raised in cases of gut pre-cancerous (IBDs and IBS) and cancerous lesions^12–15^. The A/B ratio is also increased in metabolic diseases such as type 2 diabetes and obesity^17^.

The gut-lung axis is bidirectional, and the communication pathway can be direct (via the oral cavity or sputum) or indirect, involving the bloodstream in relation to the gut bacteriome. SCFAs are also beneficial for inflammatory conditions of the lung via bone marrow hematopoietic precursors and circulating monocytes/neutrophils, enhancing their regulatory and anti-inflammatory programming. They also signal via airway epithelial and immune cells (e.g., alveolar macrophages, Tregs) through GPR41/43 and HDAC inhibition to promote barrier integrity and dampen lung inflammation^18^. While both the bacterial gut metagenome^7^ and metatranscriptome^19^ have been recently linked to COPD, the gut mycobiome and its ecological network with bacteria have not yet been elucidated.

In this study, we aim to analyze the gut mycobiome in non-small cell lung cancer (NSCLC) patients according to their COPD status with ITS sequencing. Moreover, we utilized the metagenome data from our earlier validation cohort^21^ to create bacterial-fungal co-occurrence networks and to investigate ecological hubs and rewiring. Our approach aims to provide novel insights into the gut–lung axis by characterizing fungal bacterial interactions in COPD.

## Methods

### Study population

A total of 61 patients diagnosed with stage IV NSCLC and receiving standard-of-care therapy approved by the Institutional Oncology Team were enrolled in our study cohort between 2017 and 2020 at the County Hospital of Pulmonology, Torokbalint, Hungary. Clinicopathological data included age, gender, smoking pack year (PY), and COPD Global Initiative for Chronic Obstructive Lung Disease (GOLD) stage at the time of lung cancer diagnosis. Patients were classified as COPD severe (GOLD stadium C and D) and COPD mild / no COPD (GOLD A and B and no comorbidity). All patients were assessed with an Eastern Cooperative Oncology Group (ECOG) performance status of 0–1 at the time of fecal sampling. Before sampling, all COPD patients received standard-of-care therapy contemporary guidelines in accordance with the current GOLD guidelines. Patients receiving systemic antibiotic therapy or an acute exacerbation within 30 days of fecal sampling were excluded from the study cohort. Inclusion/exclusion criteria and clinicopathological data of the study cohort are shown in Tables 1 and 2.

### Internal transcribed sequencing (ITS)

For fungal community profiling, ITS2 amplicon sequencing was performed on genomic DNA extracted from stool samples of lung cancer patients stratified by COPD status. DNA concentration was measured using a Qubit fluorometer, and 200 ng DNA was used as PCR input for each sample. The ITS2 region was amplified using fungal-specific barcoded primers, and purified amplicons were pooled in equimolar amounts to generate the sequencing library. Libraries were prepared using a standard Illumina-compatible workflow and sequenced on an Illumina HiSeq 2500 platform. Raw reads were demultiplexed and quality filtered, followed by taxonomic assignment to generate sample-wise fungal abundance tables for downstream analysis. For genus-level analyses, taxonomically assigned fungal reads/features sharing the same genus annotation were aggregated within each sample to obtain a genus-level abundance matrix. These genus-resolved profiles were subsequently used for diversity, differential abundance, ecological guild, and network-based analyses.

### Metagenomic sequencing

For bacterial abundances, we used metagenomic shotgun sequencing data from *n* = 47 patients overlapping with the ITS2 cohort, corresponding to the validation cohort of our previously published study^22^. High-quality reads were taxonomically profiled using MetaPhlAn2 (version 2.7.7) with default parameters to generate relative abundance estimates at multiple taxonomic levels. For the current analysis, genus-level abundances were extracted and matched with corresponding fungal genus-level profiles from ITS2 sequencing. Only taxa present in at least 10% of samples and with a minimum of 1% total abundance threshold were retained. The merged bacterial–fungal abundance table served as the input for cross-kingdom correlation analyses and network construction.

### Diversity analyses

Alpha-diversity was quantified with three complementary indices calculated in phyloseq v1.44: Chao1, Shannon entropy, and Simpson. For each index, values were compared between COPD-severe and mild or no-COPD groups using Welch’s test; p-values < 0.05 were considered significant. Rarefaction curves confirmed saturation of all metrics at the chosen depth.

To capture overall compositional turnover, we used a partial-least-squares (PLS) distance as a single surrogate for conventional β-diversity measures. Briefly, before CLR transformation, a pseudocount of 0.01 was added to the genus- and species-level abundance tables to handle zero values. The resulting centred log-ratio (CLR)–transformed genus and species tables were subjected to PLS regression with COPD status (severe vs. mild or no COPD) as the response in mixOmics v6.22. The first two latent components (PLS1 + PLS2) explained 65% of the between-sample variance in the species data and 69% in the genus data, respectively. A Euclidean distance matrix was then calculated based on these component scores; this “PLS distance” integrates both compositional dissimilarity and group-discriminative structure and has been shown to parallel the Bray–Curtis distance while maximizing clinical separability^22^. Group centroid separation was tested with PERMANOVA. All analyses were run in R 4.3.

### ANCOM analysis

ANCOM analysis was performed to identify differentially abundant fungal taxa between COPD non-severe (0) and COPD severe (1) groups. The abundance data were first filtered to exclude samples without COPD Severe classification. Total Sum Scaling (TSS) normalization was applied to mitigate compositional bias. For genus-level analysis, data were aggregated by summing abundances across species. A Wilcoxon rank-sum test was conducted for each taxon, and Benjamini-Hochberg FDR correction was applied to account for multiple testing. ANCOM detects subtle compositional differences by leveraging log-ratio transformations across all taxa pairs, capturing consistent shifts that absolute or relative abundance comparisons may overlook. This framework enhances sensitivity to nuanced community changes, even when individual taxa vary modestly, making it well-suited for distinguishing subtle microbiome alterations ^23,24^.

### Fungal ecology

Genera-resolved ecological profiles were visualised with a two-layer sunburst diagram. First, the top 40 abundant genera, regardless of patient group, were retained. Each genus was annotated to a primary ecological guild using the FUNGuild v1.3 reference database (confidence ≥ “probable”); unresolved taxa were labelled “Undefined Saprotroph”. Guild names formed the inner ring, and genera formed the outer ring. Abundances were centre-log-ratio normalised, converted to relative percentages, and exported to Plotly 5.20. To compare guild composition between COPD-severe and mild or no COPD patients, each sample’s genera were collapsed to the guild level, summed, and expressed as a percentage of total ITS reads. Medians for the two patient groups were plotted. Statistical differences were screened with two-tailed Wilcoxon rank-sum tests (α = 0.05).

### Correlation and network analysis

Spearman pairwise correlations were computed and edges with |ρ| > 0.4 and Benjamini–Hochberg-adjusted *p* < 0.05 were retained to build an undirected weighted graph in networkx; node size scaled to degree and edge width to |ρ|. Separate matrices were generated for COPD-severe and mild or no COPD samples, and two subnetworks were rendered with identical layout seeds. Betweenness and closeness centralities were calculated for each genus in both graphs; shifts (Δcentrality = value₁ – value₀) were exported, ranked, and visualised as lollipop plots (matplotlib) and spring-layout differential networks (red = stronger, blue = weaker in COPD severe).

The cleaned ITS table (fungi) and the MetaPhlan genus table (bacteria) were vertically concatenated after CLR transformation and aligned on 47 overlapping samples. Inter-kingdom Spearman correlations were computed; only bacteria–fungus pairs with |ρ| > 0.4 were retained. Guild information for fungi (FUNGuild) and the phylum for bacteria coloured nodes in the network visualization. Fisher-z statistics (n₀ = 27, n₁ = 20) were calculated for each edge to compare COPD groups; Δz and raw p values provided an edge-rewiring score. Edges with *p* < 0.05 formed a rewiring network whose line thickness scaled with |Δρ|. In parallel, the degree was recalculated at |ρ| > 0.4 for each genus in both groups; Δdegree values were sorted and the top 30 taxa plotted as colour-coded lollipops (purple = fungi, green = bacteria). A second lollipop summarised the 30 strongest rewired pairs (largest |Δz|). All correlations used pair-wise complete ranks.

### Supervised compositional balance analysis

ITS and metagenome-derived abundances (*n* = 47) were offset with a pseudocount (0.5) and log-transformed; the balance score for each sample was defined as the difference between the mean log-abundance of a numerator set and a denominator set of genera (i.e., log of the ratio of geometric means). We identified these sets via a greedy forward selection (selbal-like): starting from the best discriminating 1-vs-1 pair for COPD-severe (1) vs. mild or no COPD (0) and iteratively adding genera to the numerator or denominator when cross-sectional AUC improved by ≥ 0.01, with a cap of eight genera total to limit model complexity. Performance was summarised by the ROC AUC with bootstrap 95% CI (1,000 resamples) and an exact permutation test (labels shuffled 2,000 times) using a fixed random seed. Stability was assessed by repeated stratified resampling (20 half-splits), recording the selection frequency of each genus in the numerator/denominator. The analysis was implemented in Python (pandas, scikit-learn, matplotlib). The code used for the supervised compositional balance analysis is publicly available at GitHub (https://github.com/doradavid11/copd-gut-mycobiome-balance-classifier.git).

## Results

Fecal ITS sequencing data were available for *n* = 61 lung cancer patients diagnosed with severe COPD (Gold C-D stadium) or non-severe, defined as mild / no COPD (Gold A-B or no COPD comorbidity), who were included to conduct this study. After excluding patients (*n* = 4) who received systemic antibiotic treatment within 30 days of sampling, 57 patients were analyzed. Patients were categorized as having severe COPD (*n* = 18), or non-severe COPD (*n* = 39). Table 1 shows population screening, subsets and eligibility criteria, whereas Table 2 displays clinicopathological characteristics of the ITS cohort.

**Table 1:** Population screening, eligibility, and analytical subsets.

Screening step	*n*	Details
Patients enrolled in NSCLC cohort (2017–2020)	61	Advanced NSCLC cohort enrolled at the County Hospital of Pulmonology, Törökbálint.
Excluded before fungal ITS analysis	4	Exclusion criteria included recent systemic antibiotic treatment within 30 days of fecal sampling; acute exacerbation within 30 days was also an exclusion criterion.
Included in fungal ITS analysis	57	Final cohort used for fungal diversity, composition, ecological, and fungal network analyses.
Non-severe COPD group	39	Defined as GOLD A–B or no COPD comorbidity.
Severe COPD group	18	Defined as GOLD C–D.
Included in overlapping ITS + metagenome subset	47	Final matched subset used for cross-kingdom bacterial–fungal network analyses and compositional balance modeling.

Note. COPD severity grouping followed the GOLD-based clinical classification used in the study. ITS, internal transcribed spacer sequencing; NSCLC, non-small cell lung cancer.

**Table 2:** Demographic and clinicopathological characteristics of the analyzed ITS cohort stratified by COPD severity.

Characteristic	Overall (*n* = 57)	Non-severe COPD (*n* = 39)	Severe COPD (*n* = 18)	*p*-value
Male sex, n (%)	34/57 (59.6%)	22/39 (56.4%)	12/18 (66.7%)	0.567
Adenocarcinoma, n (%)	31/52 (59.6%)	22/34 (64.7%)	9/18 (50.0%)	0.378
Squamous histology, n (%)	21/52 (40.4%)	12/34 (35.3%)	9/18 (50.0%)	0.378
PD-L1 positive, n (%)	9/57 (15.8%)	9/39 (23.1%)	0/18 (0.0%)	0.045*
Prior chemotherapy recorded, n (%)	56/57 (98.2%)	38/39 (97.4%)	18/18 (100.0%)	1.000
Antibiotic use history, n (%)	11/55 (20.0%)	6/37 (16.2%)	5/18 (27.8%)	0.473
BMI ≥ 30 kg/m², n (%)	18/52 (34.6%)	13/35 (37.1%)	5/17 (29.4%)	0.758
Stage IV (metastatic), n (%)	45/57 (78.9%)	31/39 (79.5%)	14/18 (77.8%)	1.000

Note. Values are shown as n/N (%). Histology was available for 52 patients, antibiotic-use history for 55 patients, and BMI dichotomy for 52 patients. BMI dichotomy was based on a threshold of 30 kg/m². Disease stage was collapsed into stage IV (metastatic) versus earlier-stage advanced/non-metastatic disease. P-values were calculated using Fisher’s exact test.

### Mycobiome diversity and composition according to COPD status

**Fig. 1:**
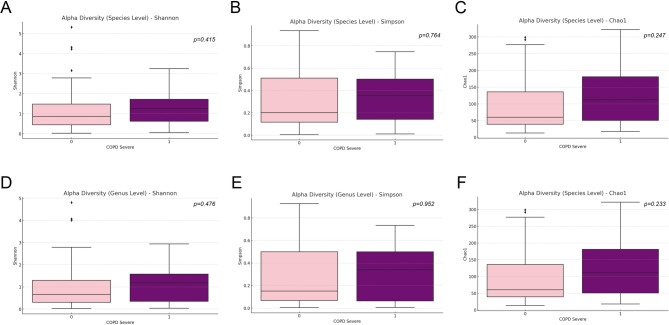
Alpha diversity of the gut mycobiome according to COPD. Shannon, Simpson and Chao1 alpha diversity metrics were calculated for patients with severe COPD (1), and no- or mild COPD (0) at species and genus levels for gut mycobiota (A-F). Statistical comparison of diversity indices were performed using Welch’s test. Statistical significance *p < 0.05; **p < 0.01, ***p < 0.001, all p-values were two-sided.

Alpha diversity was measured at the level of fungal species and genera, where we compared Shannon (Fig. 1A, D), Simpson (Fig. 1B, E), and Chao1 (Fig. 1C, F) indices between the COPD severe (1) and COPD mild / no COPD (0) groups, where none of the comparisons showed a significant difference.

**Fig. 2:**
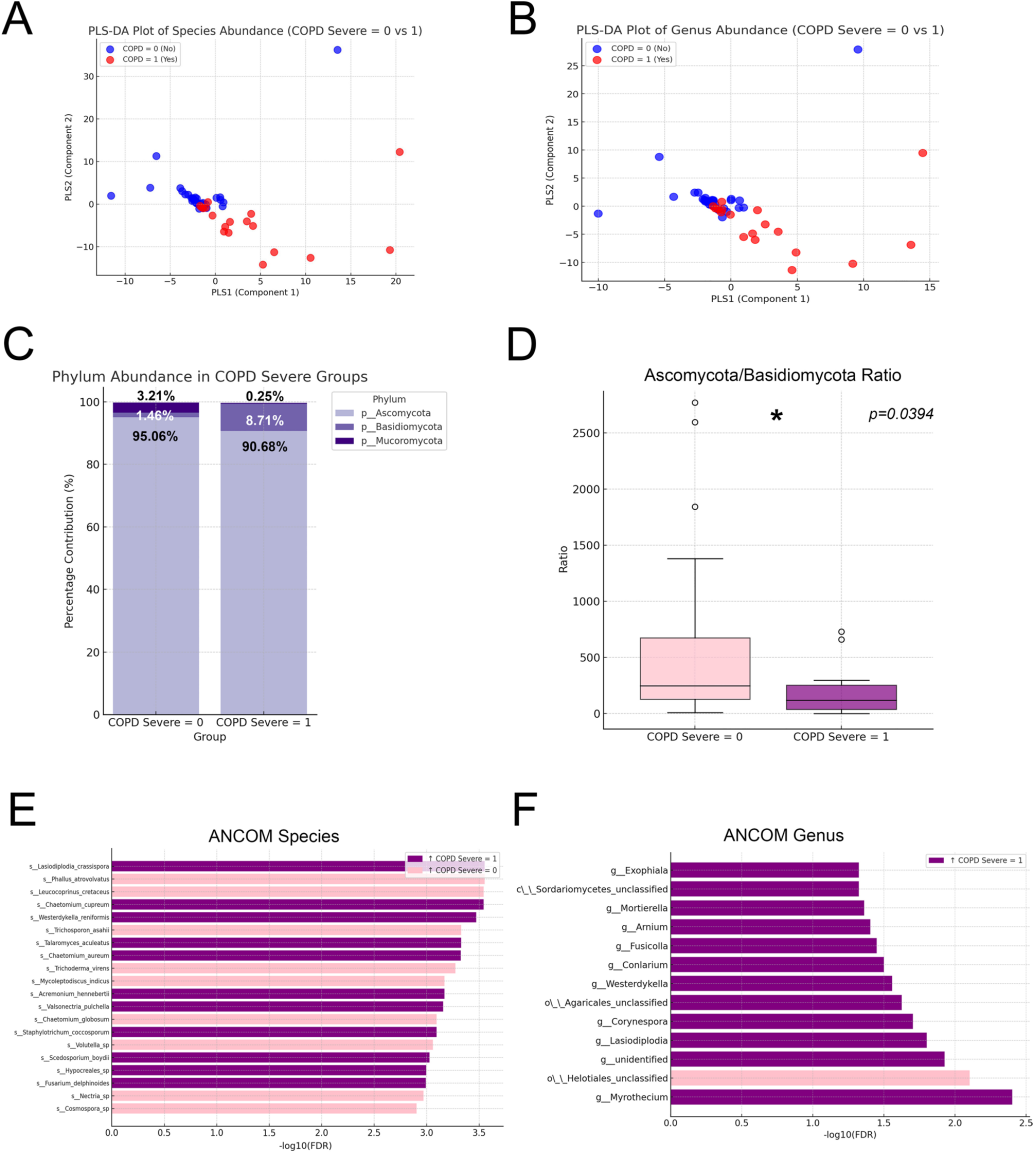
Compositional differences of the gut mycobiome according to COPD. Partial least squares discriminant analysis (PLS-DA) performed on abundance data is shown at the species (**A**) and genus (**B**) levels. Axes represent the first two latent components that maximize between-group variance, demonstrating partial but reproducible separation despite overlap. Group differences were evaluated using PERMANOVA on the PLS distance matrix, yielding a borderline significant effect of COPD status (pseudo-F = 2.1, R² = 0.06, *p* = 0.058). Phylum-level profiles (C) illustrate relative contributions of Ascomycota, Basidiomycota, and Mucoromycota, with values expressed as mean percentage abundance across groups. The Ascomycota/Basidiomycota ratio (D) was visualized using boxplots, confirming a significant reduction in severe COPD (*p* = 0.0394). Differential abundance was further examined using Analysis of Compositions of Microbiomes with Bias Correction (ANCOM), a log-ratio framework that accounts for compositionality and controls false discovery. Results are displayed for species (E) and genera (F), ranked by −log10(FDR). ANCOM significance threshold was set to −log10(FDR) > 2. Bars indicate directionality, with purple denoting taxa overrepresented in COPD-severe and pink indicating enrichment in non-severe patients.

Next, we aimed to assess whether there was a significant compositional shift between patients with severe vs. non-severe COPD. A sensitive analysis, Partial Least Squares Discriminant Analysis (PLS-DA) was used to reveal subtle differences in composition data between the two patient groups. PLS-DA maximizes the separation between the two groups by finding the optimal combination of taxa that best differentiates them. Although there was some overlap (Fig. 2A-B), PLS-DA revealed a separation pattern linked to COPD status among genera and species (Fig. 2A-B). Permanova on the PLS distance matrix yielded a borderline-significant effect of COPD status (pseudo-F = 2.1, R² = 0.06, *p* = 0.058), indicating that 6% of overall fungal community variance is attributable to disease severity.

Next, we compared the fungal macrocomposition of the two patient groups, listing key phyla and the ratio of *Ascomycota* to *Basidiomycota* (A/B ratio). The relative abundance of *Ascomycota* (90.68% vs. 95.06%) to *Basidiomycota* (8.71% vs. 1.46%) was significantly lower in patients with severe COPD (vs. non-severe COPD, *p* = 0.0394, Fig. 2C-D). In addition, the abundance of phylum *Mucoromycota* also decreased in patients with severe COPD compared to patients with mild or no COPD (0.25% vs. 3.21%).

Analysis of Compositions of Microbiomes with Bias Correction (ANCOM) was performed to identify differentially abundant fungal taxa between the two clinical patient groups. ANCOM identified genera *Myrothecium* (overrepresented in severe COPD) and Helotiales_unclassified (overrepresented in mild or no COPD) with an above-log10 (FDR) value of 2. At the species level, the top signals included *Lasiodiplodia crassispora* (enriched in COPD-severe) and *Phallus atrovolvatus* (enriched in non-severe). Other species enriched in severe cases were *Trichosporon asahii*, *Talaromyces aculeatus*, and *Chaetomium aureum*. In contrast, species such as *Leucocoprinus cretaceus*, *Trichoderma virens*, and *Cosmospora sp.* were more abundant in patients with non-severe conditions.

### Correlation of fungal genera and fungal networks in COPD

**Fig. 3:**
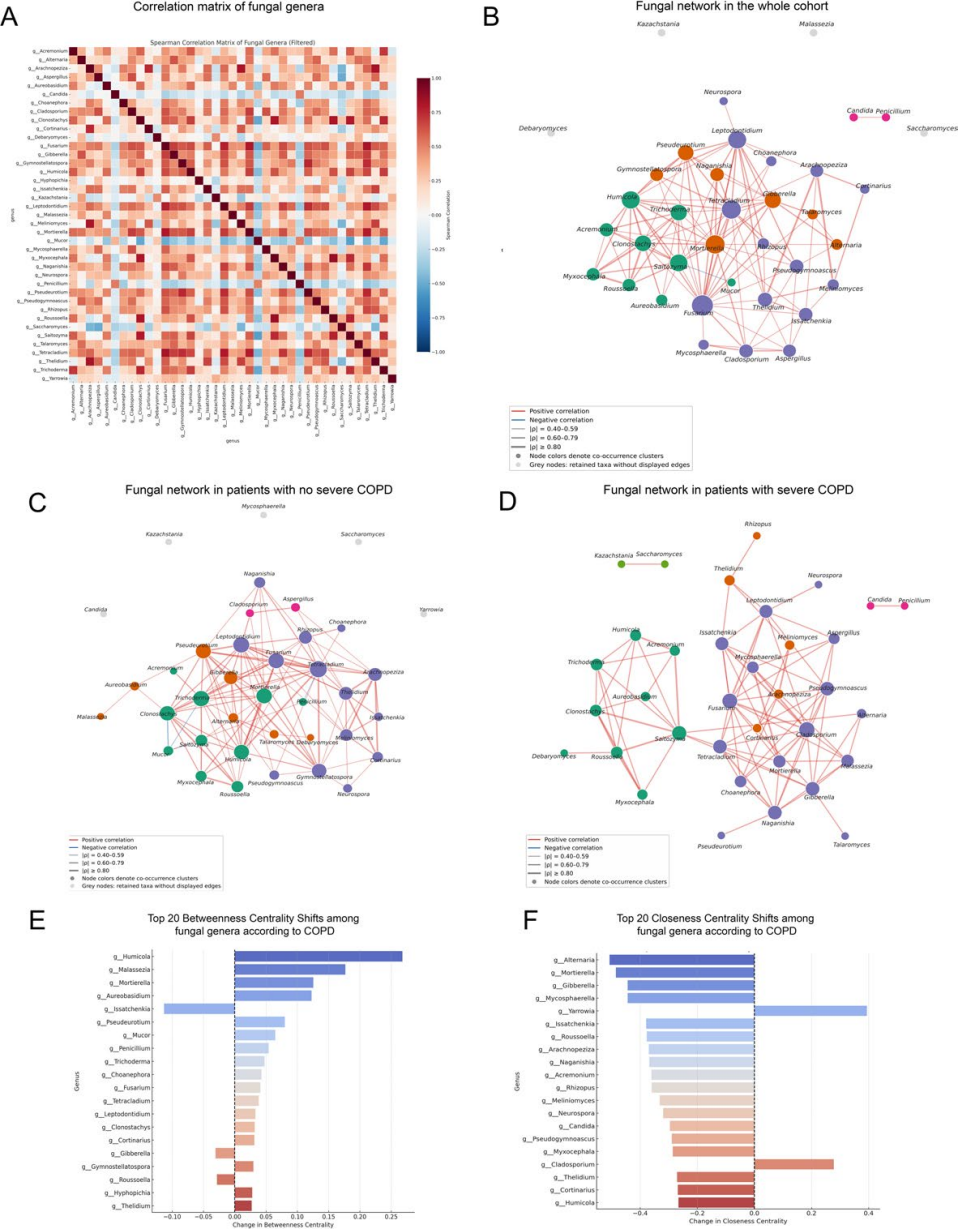
Correlation of fungal genera and the gut mycobiome network in COPD. Panel A shows the Spearman correlation matrix of 40 most abundant fungal genera. After filtering for |ρ| > 0.4 and *p* < 0.01, a weighted undirected gut mycobiota network is dominated by positive correlations shown in panel B. Panels C and D present stratified networks for patients with and without severe COPD, respectively. The non-severe network exhibited higher modularity and a greater proportion of strong positive correlations, while the severe network was more fragmented and included several negative associations. Red and blue colored edges depict positive and negative correlations, respectively. The size of each node is proportional to its degree in the network (the number of connections it has), shared node colors classify correlating genera to co-occurrence clusters. Panels E and F show the top 20 shifts in betweenness and closeness centrality, respectively. Positive bars show genera more central in patients with severe COPD, whereas negative bars show genera more central in patients with no severe COPD.

We constructed and analyzed genus-level co-occurrence networks derived from the fungal ITS data, stratified by the presence or absence of severe COPD. We applied Spearman correlation analysis (Fig. 3A), retaining only robust associations (|ρ| > 0.4, FDR-adjusted *p* < 0.01) to construct a weighted, undirected network that included all patients (Fig. 3B). The ITS genus–genus matrix was dominated by positive co-occurrences. ρ ≥ 0.8 pairs were: *Myxocephala–Roussoella* (ρ = 0.86), *Fusarium–Tetracladium* (ρ = 0.82), *Arachnopeziza–Thelidium* (ρ = 0.82), *Arachnopeziza–Meliniomyces* (ρ = 0.81), *Clonostachys–Roussoella* (ρ = 0.8), *Clonostachys–Myxocephala* (ρ = 0.8), *Leptodontidium–Tetracladium* (ρ = 0.8), *Fusarium–Mortierella* (ρ = 0.8). The remaining 60 pairs with ρ = 0.60–0.8 repeatedly linked plant/soil and litter-associated genera (*Debaryomyces*,* Kazachstania*,* Cladosporium*,* Aspergillus*,* Penicillium*) with root- or wood-associated saprotrophs/mycoparasites (*Leptodontidium*,* Trichoderma*,* Tetracladium*,* Clonostachys*,* Mortierella*). In contrast, no negative correlations exceeded |ρ| = 0.6; the strongest antagonistic trends were *Mucor–*Saitozyma (ρ=−0.545), Clonostachys*–Mucor* (ρ=−0.512), *Mucor–Trichoderma* (ρ=−0.508), *Aspergillus–Saccharomyces (ρ=−0.498).* Collectively, the signal suggests co-varying environmental guilds—wood/leaf-litter saprotrophs, mycoparasites, and root-associated fungi—move together in the gut (likely reflecting shared sources or trophic coupling), while *Mucorales* show niche exclusion with filamentous *Ascomycetes* and basidiomycetous yeasts.

Separate networks were generated for patients without severe COPD (*n* = 39) and with severe COPD (*n* = 18). The non-severe COPD network exhibited higher complexity, comprising 33 fungal genera connected by 64 significant edges, with a network density of 0.121, average degree of 3.88, and average clustering coefficient of 0.403. In contrast, the severe COPD network was sparser, with only 28 nodes and 39 edges (density: 0.101; average degree: 2.79; clustering coefficient: 0.329). These differences indicate reduced co-occurrence connectivity in patients with severe COPD. Edge-level comparison revealed that only 21 correlations were shared between groups, while 43 and 18 were unique to the COPD = 0 and COPD = 1 networks, respectively. The COPD = 0 network contained a greater proportion of strong positive associations, suggesting network stability, whereas the COPD severe network included several negative correlations, indicative of potential antagonistic interactions or niche exclusion.

Modularity analysis further demonstrated that the non-severe COPD network had more distinct and interconnected community modules, whereas the severe COPD network showed reduced modularity and fewer hub taxa. Collectively, these findings suggest that severe COPD is associated with a less complex and more fragmented structure of fungal co-occurrence. Altogether, the COPD-severe-associated network is characterized by a reduction in complexity, loss of connectivity, and a shift toward more isolated interactions among fungal taxa.

### Gut fungal ecology and the bacteria-fungal network in COPD

**Fig. 4:**
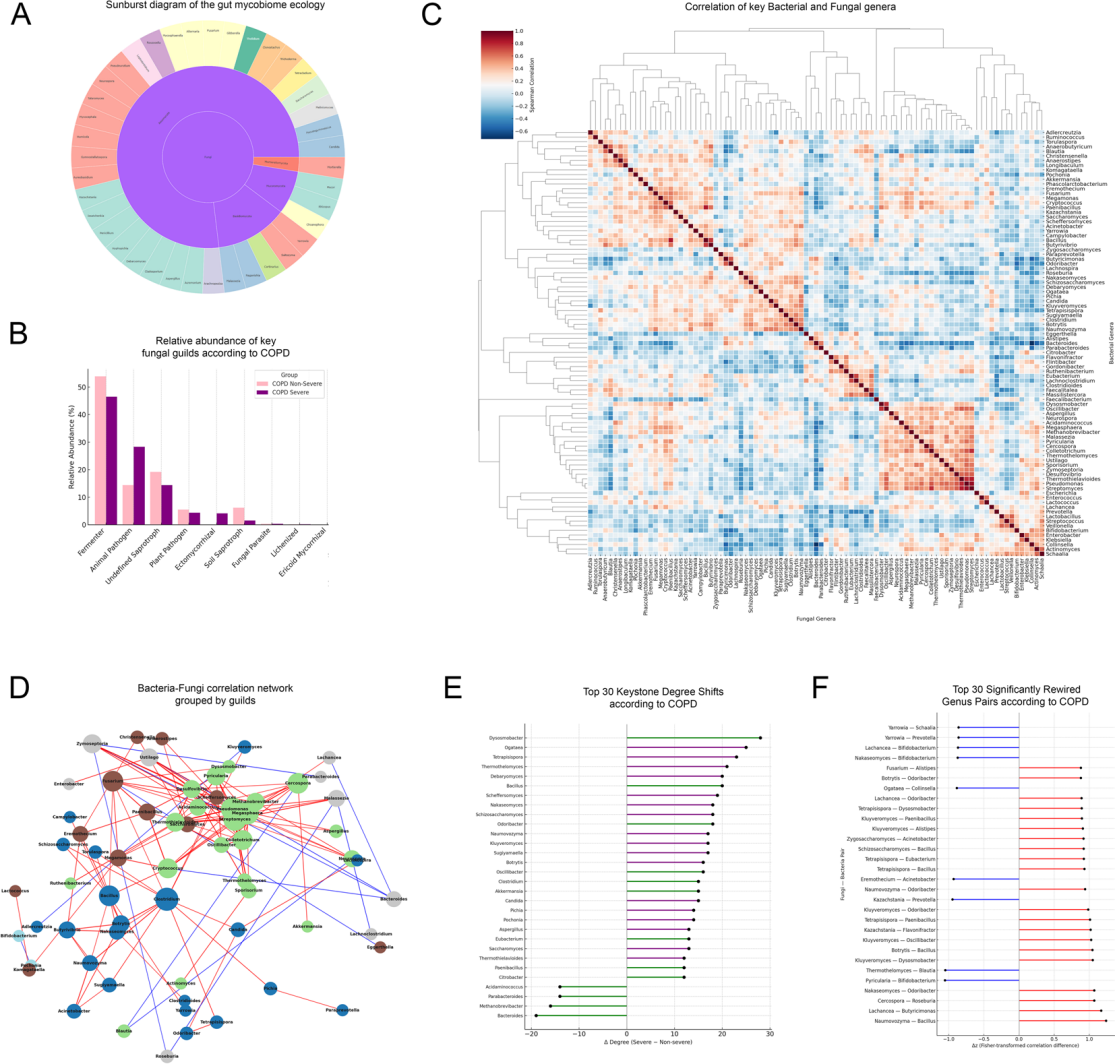
Cross-kingdom ecology and co-occurrence network perspective of the gut mycobiome in COPD. Panel A shows a sunburst diagram of the gut mycobiome at the phylum and genus level, with outer segments color-coded by ecological guild. The relative abundance of major fungal guilds across patient groups is shown in a bar chart (B), indicating significant enrichment of animal pathogens and ectomycorrhizal fungi in COPD-severe patients. At the same time, soil saprotrophs were more abundant in non-severe cases. Panel C presents a hierarchical clustered correlation heatmap of bacterial and fungal genera derived from overlapping metagenome and ITS datasets, including the 126 significant associations with Spearman correlation |ρ| > 0.4 and *p* < 0.05. Distinct modules are visible, with clusters of bacterial SCFA producers (Ruminococcus, Eubacterium, Butyricicoccus) showing negative correlations with fungal pathobionts (Kazachstania, Malassezia), whereas Candida, Debaryomyces, and Saccharomyces align with pro-inflammatory bacterial taxa such as Enterococcus, Escherichia/Shigella, and Klebsiella. Panel D depicts the bacteria–fungi correlation network, organized by guilds into four ecological communities: a fermentative dysbiosis niche (brown), an anaerobic SCFA-rich core (blue), a soil-derived immunomodulator cluster (green), and a commensal axis containing Bacteroides, Parabacteroides, Roseburia, and Malassezia (gray). Node sizes represent abundance, and edges are colored red for positive and blue for negative correlations. Lollipop charts show the top 30 keystone degree shifts between COPD-severe and non-severe patients (E), where taxa such as Candida, Aspergillus, Enterococcus, and Clostridium gained centrality, while Akkermansia and Roseburia lost centrality. The top 30 significantly rewired genus pairs, according to COPD (F), showed strengthened correlations in severe cases, concentrated around Candida and facultative anaerobes, while weakened edges predominantly involved SCFA-associated bacteria and saprotrophic fungi. X-axis represents the magnitude of network change between COPD-severe and non-severe groups: difference in keystone degree centrality values (gain or loss of connectivity importance for each genus, E), and the Δρ correlation strength for each significantly rewired bacterial–fungal genus pair (F).

To further explore the association between the gut mycobiome and COPD, we aimed to investigate which ecological niches and fungal guilds are enriched in patients with severe vs. non-severe disease. Sunburst diagram shows the taxonomic breakdown at the phylum and genus level of the gut mycobiome in our cohort in the context of major taxonomical guilds (Fig. 4A). Major ecological guilds include Plant pathogens, Lichenized fungi, Fungal parasites, Freshwater saprotrophs, Fermenters, Ericoid mycorrhizals, Animal pathogens, Soil saprotrophs, Ectomycorrhizals, Wood saprotrophs, Root endophytes, Plant saprotrophs, and unidentified saprotrophs that couldn’t be classified to neither guild. When comparing the two patient groups, we found that animal pathogens (28% vs. 14%, *p* = 0.007) and ectomycorrhizal fungi (4% vs. 0.2%, *p* < 0.001) are significantly more prevalent in patients with severe COPD. In contrast, fibre-degrading soil saprotrophs were overrepresented in mild or no COPD (7% vs. 2%, *p* < 0.032) (Fig. 4B).

To establish a correlation and network map between bacteria and fungi, we used the metagenome data-derived bacterial abundances of the 47 overlapping patients from our NSCLC cohort, published previously for validation purposes^21^. Concatenated and normalized abundances of top genera (1% cut-off) were correlated using Spearman’s rank correlation (Fig. 4C). Then, a co-occurrence network was created, adjusted for ecological guilds (Fig. 4D). The heatmap and hierarchical clustering revealed distinct co-occurrence modules. Notably, some fungal taxa (*Saccharomyces*, *Debaryomyces*, *Candida*) tend to cluster with bacterial genera typically found in disturbed or inflamed gut environments (e.g., *Enterococcus*, *Escherichia*/*Shigella*, *Klebsiella*, and *Clostridium*), suggesting a possible shared association with gut dysbiosis or environments related to inflammation. Clusters of bacterial genera involved in fiber degradation and SCFA production—such as *Ruminococcus*, *Eubacterium*, and *Butyricicoccus*—appear inversely related to fungi typically classified as pathobionts or stress-tolerant (e.g., *Kazachstania*, *Malassezia*). Several fungal genera show consistent anti-correlation with taxa known to maintain gut homeostasis (*Faecalibacterium*, *Blautia*, *Roseburia*).

Co-occurrence analysis revealed four major cross-kingdom ecological communities: a fermentative dysbiosis niche, that combines probiotic and fermentative bacteria with ascomycetous yeasts and plant-associated fungi (*Lactococcus*, *Paenibacillus*, *Saccharomyces*, *Fusarium*, *Anaerostipes*, brown, Fig. 4D); an anaerobic Core and gut barrier modulator niche with abundant SCFA producers (*Clostridium*, *Blautia*), co-occurring or negatively correlated with fungal pathobionts like *Candida* (blue, Fig. 4D); a community of soil-derived immunomodulators, genera involved in secondary metabolite production and often found in inhaled particles or soil-contaminated foods (*Streptomyces*, *Cryptococcus*, *Actinomyces*, *Dysosmobacter*, *Thermothelymices*, green, Fig. 4D); and a default commensal axis including key commensal, high-abundance anaerobes and low-abundance environmental fungi (*Bacteroides*, *Parabacteroides*, *Roseburia*, *Malassezia*, gray, Fig. 4D).

In the case of the Keystone analysis, centrality increased for *Candida*, *Aspergillus*, *Enterococcus*, and *Clostridium*, while it decreased for *Akkermansia* and *Roseburia*. These results indicate a shift from fiber-associated, butyrate-linked networks toward oxygen-tolerant, fermentative/proteolytic consortia in COPD-severe cases (Fig. 4E). Cross-kingdom edge rewiring showed more edges strengthening in COPD-severe than weakening. Stronger correlations concentrated on the yeast *Candida* and facultative anaerobes *Enterococcus* and *Clostridium*, with additional positive links to *Akkermansia* and *Lactobacillus*. Weaker correlations centered on SCFA-associated taxa (*Faecalibacterium*, *Roseburia*) and saprotrophic fungi (*Debaryomyces*, *Saccharomyces*) (Fig. 4F).

### Predictive function of compositional balance

**Fig. 5:**
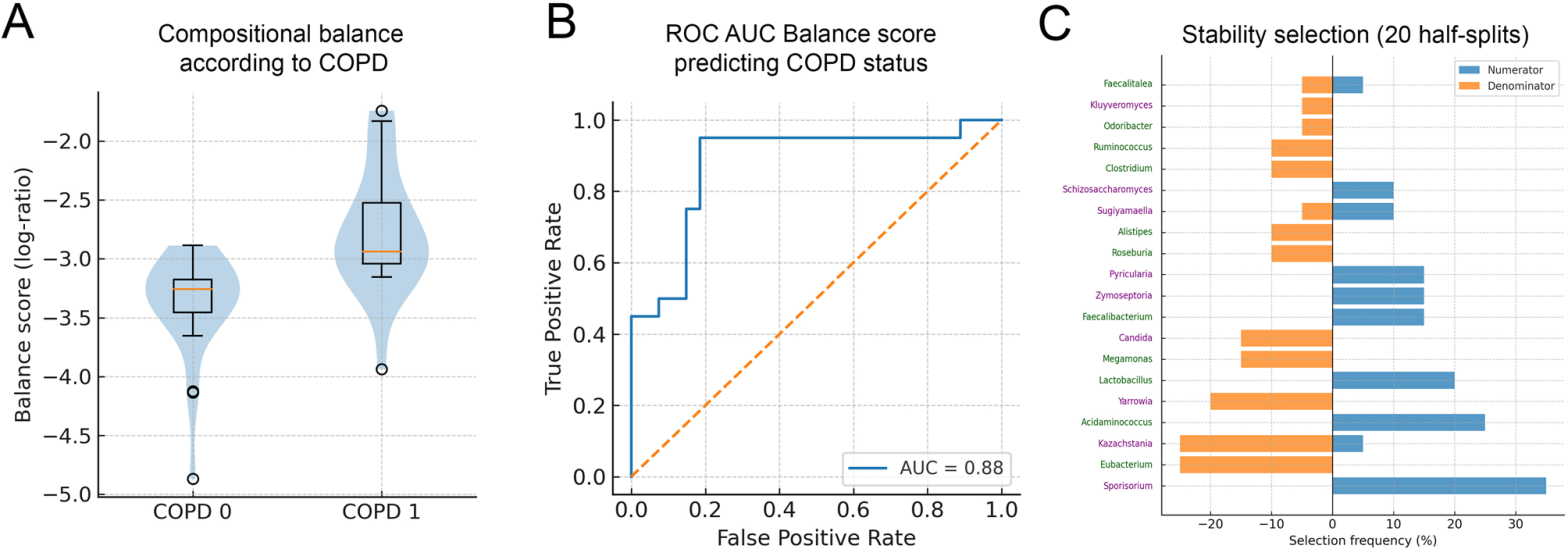
Predictive function of cross-kingdom co-occurrence niches. Balance score is the difference between the mean log-abundance of a numerator set and a denominator set of genera (score = mean[log numerator] − mean[log denominator]; pseudocount 0.5). Sets were selected using a greedy forward procedure seeded with the best 1-vs-1 pair and expanded until the AUC improved by ≥ 0.01, capped at a total of eight genera. (**A**). Performance was summarised by the ROC AUC with bootstrap 95% CI (1,000 resamples) and an exact permutation test (labels shuffled 2,000 times) using a fixed random seed (**B**). Stability was assessed by repeated stratified resampling (20 half-splits), recording the selection frequency of each genus in the numerator/denominator (**C**).

We used a supervised compositional balance classifier to derive a single, interpretable per-patient score that contrasts “pro-severe COPD” versus “anti-severe COPD” genera according to COPD. This method is suitable for microbiome data (which are inherently relative), avoids spurious correlations, and directly tests the network-level hypothesis of hub replacement. In the 47 matched ITS and metagenome sequenced samples, higher scores were observed in COPD-severe (Fig. 5A), yielding strong discrimination (AUC = 0.88 with restricted candidates; 0.93 when all genera were allowed, Fig. 5B). The balance selected *Sporisorium* in the numerator and *Alistipes*, *Eubacterium*, *Kazachstania*, and *Kluyveromyces* in the denominator, and significance by permutation. Stability resampling repeatedly recovered the same pattern (*Sporisorium* as the most frequent numerator; *Alistipes*/*Eubacterium*/*Kazachstania*/*Kluyveromyces* as denominators), indicating a robust direction of effect even if individual taxa enter with moderate frequencies (Fig. 5C). Biologically, the balance captures a tilt toward opportunistic fungi relative to commensal, fermentation-linked bacteria/yeasts, consistent with our network findings: strengthened co-occurrence around aerotolerant, inflammation-adapted taxa and weakening of SCFA-associated partners.

## Discussion

Despite comprising a small share of the gut ecosystem, fungi deliver potent cell-wall ligands and metabolites that reset mucosal immunity and propagate signals along the gut–lung axis [23, 10, 6]. In COPD, gut-derived molecular signals can alter exacerbation risk and modulate responses to infection and therapy^10,7^. When fungal communities rewire cross-kingdom networks and erode SCFA–centered trophic chains, barrier instability and systematic inflammation can follow, amplifying lung pathology. Conversely, network reconstitution—via diet, prebiotics, or targeted microbiome strategies—might alleviate disease. Our data, therefore, position the gut mycobiome as a context-dependent biomarker of pulmonary homeostasis and severe COPD, with implications for risk stratification and future microbiome-based interventions.

In the current study, we profiled the gut mycobiome in patients with lung cancer and those with and without severe COPD, and integrated bacterial metagenome data from the same cohort. Alpha-diversity showed no significant differences in the two patient groups, but composition and structure shifted with COPD severity. Severe COPD showed a higher Ascomycota/Basidiomycota ratio, enrichment of opportunistic fungi, and a simpler, less connected fungal network. Cross-kingdom correlation patterns showed stronger co-variation between *Candida*/*Aspergillus* and Enterobacteriaceae-associated taxa, while correlations involving SCFA-associated bacteria and commensal yeasts were weaker. Overall, our results support a network “rewiring” rather than the effects of differentially abundant single-taxon species.

In our cohort, fungal alpha diversity did not differ across COPD categories (severe vs. mild or no-COPD), while composition showed a modest but reproducible separation when using PLS, a pattern is consistent with many bacterial gut studies in COPD that report limited alpha shifts but measurable community turnover^7,24^. By contrast, airway mycobiome work sometimes finds higher alpha diversity or distinct clustering in COPD—especially in frequent exacerbators—in line with a compartment-specific signal^27,28^. For stool fungi, comparative data remain scarce and variable, likely reflecting low biomass, high inter-individual variation, and technical constraints of ITS profiling. Altogether, COPD severity aligns with subtle compositional drift, not drastic diversity changes.

In phylum-level analyses, we observed a shift in the A/B ratio, with severe COPD showing relatively lower *Ascomycota* and higher *Basidiomycota*, suggesting a broad ecological tilt. Similar A/B distortions are reported in inflammatory bowel disease, GI cancers and other inflammatory states, though directionality varies across cohorts, methods, and sampling types^12,29–32^. Several studies describe the enrichment of *Basidiomycota* and the depletion of *Ascomycota* in gut inflammation, as well as in lung cancer, aligning with our pattern, while others report mixed results^33^. Biologically, an A/B shift may reflect oxygenation, bile acid, and immune pressures that favor aerotolerant or lipid-adapted *Basidiomycota* (e.g., *Malassezia*) and alter interactions with bacteria and host pattern-recognition pathways^32^. Because *Ascomycota* also contains both commensals and opportunists, the A/B ratio should be read as a coarse ecosystem marker that compresses complex mycobiome changes into a tractable metric for longitudinal assessment^12^.

Across COPD groups, fungal genus correlations reconfigured: severe COPD showed fewer and weaker positive edges, more isolated nodes, and lower modularity/robustness—hallmarks of ecosystems under inflammatory stress^34–36^. Hubs shifted toward opportunistic taxa, while commensal/food-associated yeasts lost centrality. Cross-kingdom networks mirrored this: in severe COPD, *Candida*/*Aspergillus* strengthened associations with *Enterobacteriaceae* and other facultative aerobes, whereas ties linking SCFA-producing bacteria (*Faecalibacterium* and *Roseburia*) to benign yeasts weakened or reversed. These patterns indicate network-level rewiring, not single-taxon effects. Mechanistically, inflammation-driven epithelial metabolic shifts may increase mucosal oxygen and nitrate levels, favoring facultative taxa over strict anaerobes^35,38^. Because SCFAs stabilize lung immune tone, erosion of SCFA-centered consortia offers a plausible gut–lung pathway for exacerbation-prone phenotypes^39^. Cross-kingdom studies further support fungi as active modulators rather than bystanders [38,39]. It is important to note though, that bacterial and fungal profiles were generated by shotgun metagenomic sequencing and ITS2 amplicon sequencing, respectively, so their abundance estimates are affected by distinct technical and compositional biases and should not be interpreted as directly comparable absolute measures. Therefore, the observed bacteria–fungi correlations are best understood as coordinated shifts in relative community structure within this cohort, rather than as evidence of direct ecological interactions.

COPD shows strong association with smoking (there were only two non-smokers in our cohort) and smoking-/inflammation-driven immune–trophic feedback explain our patterns. Reduced colonocyte β-oxidation and PPAR-γ signaling increase mucosal O_2_ and host-derived nitrate, favoring facultative bacteria and aerotolerant yeasts over strict butyrate producers^38^. The strengthened *Candida*–*Enterococcus*/*Clostridium* edges and weakened *Faecalibacterium*/*Roseburia*–benign yeast edges are consistent with metabolic reprogramming, characterized by lower butyrate production and enhanced lactate/ethanol/amine fluxes that reinforce inflammation and barrier stress^40–42,45^. Fecal metabolomics and further multi-omic approaches, combined with longitudinal studies, are needed to confirm these findings.

In our study, instead of classifying patients as diagnosed vs. not diagnosed with COPD, we stratified patients as severe COPD vs. mild/no COPD. Severity-based grouping more accurately reflects the biology that drives microbiome changes. Severity is associated with exacerbation burden, systemic inflammation, hypoxemia, comorbid load, and exposure to therapies, including inhaled corticosteroids and antibiotics, all of which are potent modifiers of the microbiome^28,46^. As COPD worsens, systemic inflammation rises^45^, neutrophils are more primed^46^, and hypoxemia plus catabolic stress reshape epithelial metabolism and barrier function—factors that influence gut oxygen leak, bile acids, and cross-kingdom networks^10,47,48^. Exacerbation burden also perturb the bacterial–fungal balance^51–53^. These gradients create a more precise dose–response in gut–lung signaling than a binary “COPD yes/no,” which mixes biologically near-normal mild cases with profoundly dysregulated severe cases.

Our study is limited by its cross-sectional design. Moreover, the sample size constrained multivariate and subgroup analyses; therefore, we focused on ecological and network differences rather than strict clinical or biomarker endpoints—a choice that precludes causal inference and allows residual confounding (diet, medications, inhaled corticosteroids/antibiotics, oral health). Still, converging clinical and experimental data support a reciprocal gut–lung axis in COPD: gut metabolites shape airway immunity, while pulmonary inflammation and therapies reshape gut communities^48,52^. An additional limitation is that all participants had stage IV NSCLC and were managed in a standard-of-care oncologic setting, which may itself influence the gut mycobiome through systemic inflammation, altered nutritional/metabolic status, cachexia, and treatment-related effects. Accordingly, the present findings should be interpreted as COPD-severity-associated mycobiome patterns within an advanced NSCLC cohort, rather than as broadly generalizable COPD-specific features.

## Conclusion

Taken together, our data suggest that COPD severity is mirrored not by distinct gain or loss of specific fungal taxa, but by re-wiring of cross-kingdom networks toward opportunistic, inflammation-adapted consortia and away from SCFA-producing ecosystems. Our study extends COPD microbiome work to the mycobiome and nominates network features—not single taxa—as candidate readouts to explain alteration of gut commensals in pulmonary inflammatory conditions. Prospective, multi-omics, and interventional studies should test whether restoring SCFA-centric ecological niches improves pulmonary outcomes.

## Data Availability

The datasets generated and analysed during the current study have been deposited in the NCBI sequence read archive (SRA) database under accession code PRJNA811494.
